# Improving speech perception for hearing-impaired listeners using audio-to-tactile sensory substitution with multiple frequency channels

**DOI:** 10.1038/s41598-023-40509-7

**Published:** 2023-08-16

**Authors:** Mark D. Fletcher, Carl A. Verschuur, Samuel W. Perry

**Affiliations:** 1 University of Southampton Auditory Implant Service, University of Southampton, University Road, Southampton, SO17 1BJ UK; 2 Institute of Sound and Vibration Research, University of Southampton, University Road, Southampton, SO17 1BJ UK

**Keywords:** Translational research, Engineering

## Abstract

Cochlear implants (CIs) have revolutionised treatment of hearing loss, but large populations globally cannot access them either because of disorders that prevent implantation or because they are expensive and require specialist surgery. Recent technology developments mean that haptic aids, which transmit speech through vibration, could offer a viable low-cost, non-invasive alternative. One important development is that compact haptic actuators can now deliver intense stimulation across multiple frequencies. We explored whether these multiple frequency channels can transfer spectral information to improve tactile phoneme discrimination. To convert audio to vibration, the speech amplitude envelope was extracted from one or more audio frequency bands and used to amplitude modulate one or more vibro-tactile tones delivered to a single-site on the wrist. In 26 participants with normal touch sensitivity, tactile-only phoneme discrimination was assessed with one, four, or eight frequency bands. Compared to one frequency band, performance improved by 5.9% with four frequency bands and by 8.4% with eight frequency bands. The multi-band signal-processing approach can be implemented in real-time on a compact device, and the vibro-tactile tones can be reproduced by the latest compact, low-powered actuators. This approach could therefore readily be implemented in a low-cost haptic hearing aid to deliver real-world benefits.

## Introduction

Treatment of hearing impairment has been revolutionised by the advent of cochlear implants (CIs). However, in high-income countries it is estimated that only between 5 and 13% of adults who could benefit from a CI receive one^1,2^. This is predominantly because of disorders that prevent implantation (e.g., cochlear ossification) and because of barriers in complex care pathways^3^. There is also a significant group who receive little or no benefit from their device due to factors such as long-term deafness^4^. In lower resource settings, the predominant barrier to access is insufficient healthcare provision^5^. Unmanaged hearing loss in children restricts language and cognitive development, and is associated with a lower employment rate in adulthood^5^. For older adults, unmanaged hearing loss is a significant risk factor for accelerated cognitive decline, dementia, and reduced health-related quality of life^6^.There is therefore a substantial need for a low-cost, non-invasive alternative to CI technology.

In the 1980s and 1990s, a number of sensory substitution devices were developed that provided speech cues to hearing-impaired listeners through tactile stimulation^7^. While some of these tactile aids allowed large numbers of words to be identified through tactile stimulation alone^8^ and could improve speech recognition with lip-reading by more than 15%^9,10^, by the late 1990s they were rarely used clinically. This was due to dramatic improvements in the effectiveness of CIs^7^ and to the heavily limited technology available for portable haptic devices (e.g., large batteries, highly limited signal-processing capacity, and poor haptic signal reproduction)^11^. However, dramatic advances in technology since that time mean that haptic devices could now offer a viable alternative or complement to a CI^7,11^.

A new generation of low-cost, low-powered, compact haptic actuators are able to deliver high-precision, high intensity vibro-tactile stimulation across a range of frequencies. This has been exploited in recent studies that augment the electrical CI signal with haptic stimulation (“electro-haptic stimulation”^12^), and have demonstrated substantial improvements in speech-in-noise performance^12–14^ and sound localisation^15–18^. In these studies, audio was converted to tactile stimulation using a vocoder approach. This approach converts the audio frequency range to the frequency range where tactile system is highly sensitive. To do this, the audio is first filtered into frequency bands. The amplitude envelope is then extracted for each band and used to modulate the amplitude of vibro-tactile tones. Unlike previous studies that have converted frequency to location of tactile stimulation on the skin^8,19^, this audio-to-tactile vocoder approach uses an intuitive frequency-to-frequency conversion.

Based on previous tactile^20^ and hearing^21^ studies, a single frequency band conveying the broadband amplitude envelope can provide some of the phonemic information needed for consonant identification. However, the transfer of phonemic information that is reliant on spectral cues, including that used to identify vowels, voicing, and consonant place of articulation^22,23^, will depend on the extent to which multiple frequency channels can be conveyed through tactile stimulation. Frequency difference discrimination thresholds suggest that between four and eight individual frequencies can be distinguished across the usable frequency range for the latest haptic actuators, when stimulating the wrist^24,25^. However, it is not known to what extent multiple frequency channels can be separated when presented simultaneously, and whether spectral information provided through tactile stimulation can be exploited to improve speech perception.

The current study aimed to establish whether a greater number of frequency channels allows for better tactile phoneme discrimination. Tactile stimulation was delivered to a single site on the wrist, which is a viable site for a real-world wearable haptic aid^11^. Phoneme discrimination was assessed for one, four, or eight frequency-bands and vibro-tactile tones. More frequency bands were expected to allow more phonemes to be discriminated, particularly for vowels and for consonants that differed by place of articulation or voicing, which rely heavily on spectral cues. If this multi-channel approach is found to be effective, it could be an important new means through which critical spectral speech information can be transferred in a new generation of haptic hearing aids.

## Results

**Figure 1:**
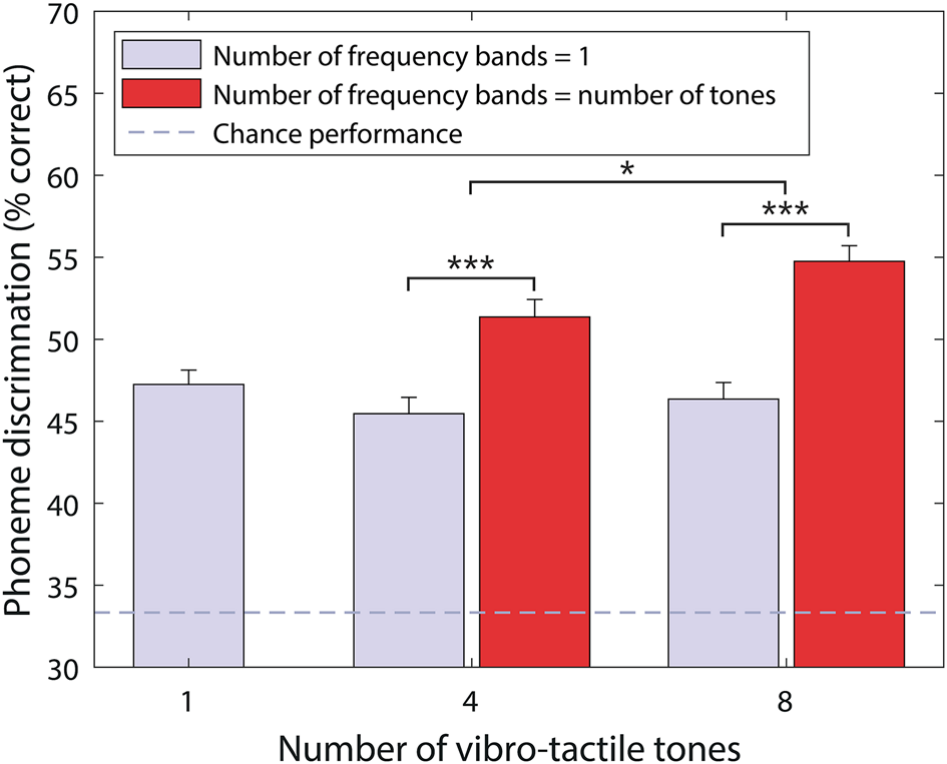
Percentage of phoneme pairs discriminated for each experimental condition, with chance performance marked by a dashed grey line. Stars show the statistical significance of differences between conditions (corrected for multiple comparisons), with more stars indicating greater significance. Error bars show the standard error of the mean (SEM).

Figure 1 shows the percentage of phonemes correctly discriminated in each experimental condition for the 26 participants who took part in this study. Primary analysis consisted of three two-tailed *t*-tests. All reported *p*-values for this primary analysis were corrected for multiple comparisons (see “Methods”). With four vibro-tactile tones, four frequency bands were found to improve phoneme discrimination by 5.9% on average (ranging from –4.3 to 17.0%; standard deviation (SD) of 5.0%) compared to one frequency band (*t*(25) = 6.0, *p* < 0.001). Performance improved from 46.5% (ranging from 38.7 to 57.5%; SD of 5.1%) to 51.4% (ranging from 39.2 to 61.8%; SD of 5.4%). With eight vibro-tactile tones, eight frequency bands were found to improve performance by 8.4% (ranging from 3.3 to 14.6%; SD of 3.0%) compared to one frequency band (*t*(25) = 14.3, *p* < 0.001). Performance improved from 46.4% (ranging from 32.5 to 57.1%; SD of 5.1%) to 54.8% (ranging from 43.9 to 64.6%; SD of 4.8%). The improvement in performance compared to baseline (one frequency band) was 2.5% larger on average for eight frequency bands than for four (ranging from –13.7 to 17.45%; SD of 5.7%; *t*(25) = 2.2, *p* = 0.035).

**Figure 2:**
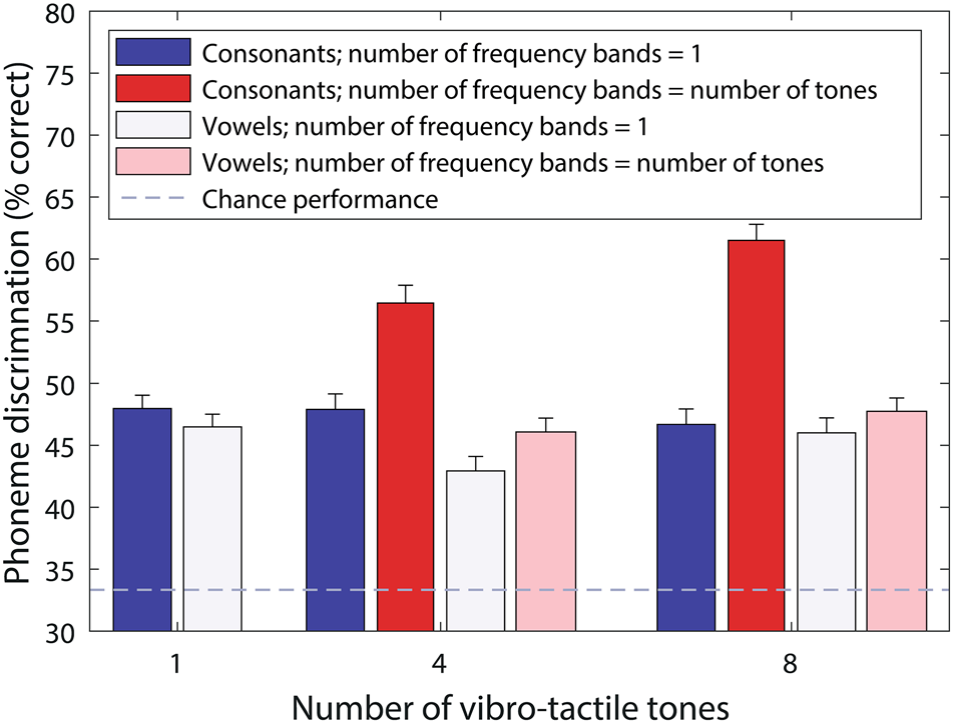
Percentage of phoneme pairs discriminated for each experimental condition, with consonant and vowel pairs shown separately. Error bars show the standard error of the mean (SEM).

The secondary analysis included multiple stages, with all the reported *p*-values for all stages corrected for multiple comparisons (see “Methods”). Figure 2 shows phoneme discrimination for consonants and vowels separately. Two two-way repeated-measures analyses of variance (RM-ANOVAs) were run on the differences between multiple-frequency-band conditions and their baselines, one for the consonants and one for the vowels, with factors ‘Number of frequency bands’ (four or eight) and ‘Talker’ (male or female). A larger improvement in performance was seen for the eight bands than for four bands for consonants (main effect of number of frequency bands: *F*(1,25) = 14.3, *p* = 0.037), but not for vowels. For consonants, with four frequency bands performance improved by 8.6% (ranging from –4.6 to 23.2%; SD of 7.6%) and with eight frequency bands performance improved by 14.8% (ranging from 7.4 to 24.1%; SD of 4.7%). For vowels, the mean performance increased by 3.1% (ranging from -5.8 to 17.3%; SD of 5.6%) with four frequency bands and by 1.7% (ranging from –7.7 to 14.4%; SD of 6.2%) for eight frequency bands. No significant main effect of talker or interaction between talker and the number of frequency bands was found for either consonants or vowels.

A three-way RM-ANOVA was then run for the baseline conditions (conditions with one frequency band), with the factors ‘Number of vibro-tactile tones’ (one, four, or eight), ‘Phoneme type’ (consonant or vowel), and ‘Talker’. No effect of the number of vibro-tactile tones was found. The overall scores with one frequency band differed by talker (main effect of talker: *F*(1,25) = 25.6, *p* = 0.001), with a mean score for the female talker of 48.7% (ranging from 37.1 to 60.4%; SD: 4.1%) and for the male talker of 44.0% (ranging from 37.4 to 54.7%; SD: 4.1%). The overall one-frequency-band scores did not differ significantly between consonants and vowels, but an interaction between talker and phoneme type was observed (*F*(1,25) = 38.4, *p* < 0.001). For the male talker, performance was 43.4% (ranging from 34.6 to 50.6%; SD of 4.5%) for consonants and 44.6% (ranging from 37.2 to 59.0%; SD of 4.9%) for vowels. For the female talker, performance was 51.7% (ranging from 38.9 to 64.8%; SD of 6.6%) for consonants and 45.7% (ranging from 35.3 to 57.1%; SD of 6.0%) for vowels.

**Figure 3:**
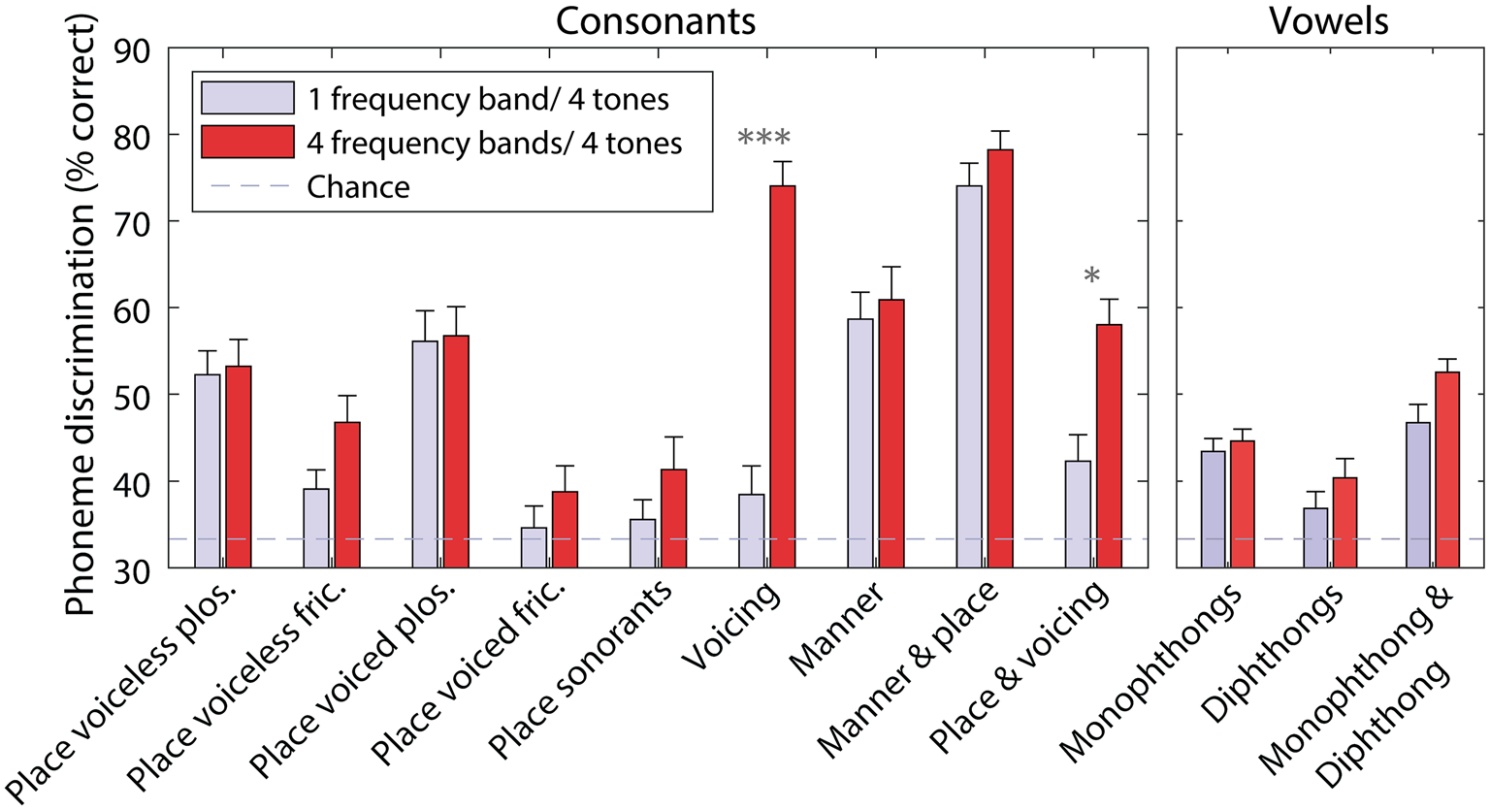
Percentage of phoneme pairs discriminated for the four-vibro-tactile-tone conditions (one or four frequency bands), grouped by phoneme contrast type. Stars show the statistical significance of differences between one and four frequency bands (corrected for multiple comparisons), with more stars indicating greater significance. Error bars show the SEM. Chance performance is marked with a dashed grey line.

Next, *t*-tests were performed to explore which phoneme contrasts were better discriminated for different numbers of frequency bands. Figure 3 shows discrimination across different phoneme contrasts for four vibro-tactile tones, with either one (baseline) or four frequency bands. Improved performance with four frequency bands was seen for consonant pairs that differed either by voicing (*t*(25) = 9.2, *p* < 0.001; mean effect: 35.6%; SD: 19.8%) or both place of articulation and voicing (*t*(25) = 3.7, *p* = 0.046; mean effect: 15.7%; SD: 21.8%).

**Figure 4:**
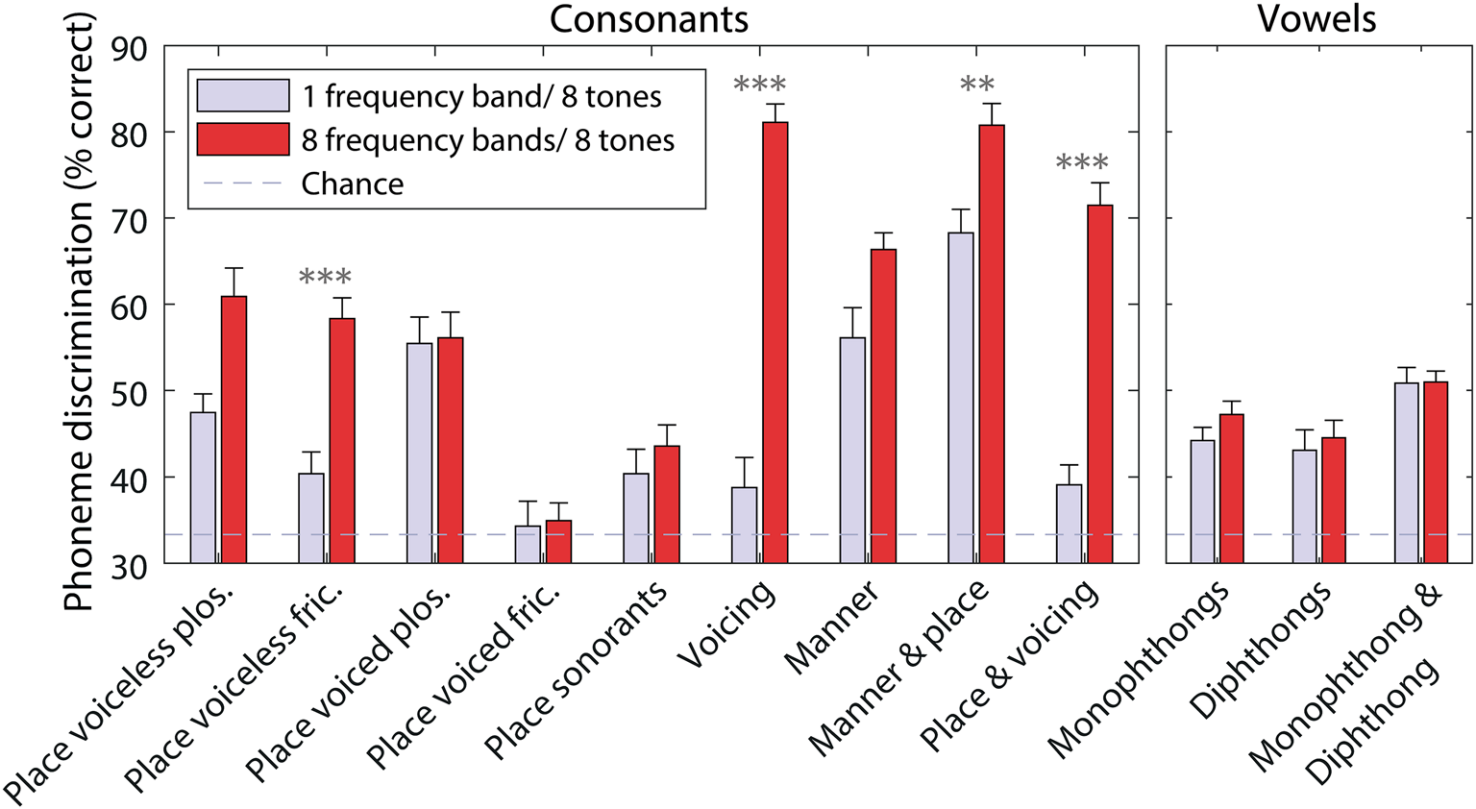
Percentage of phoneme pairs discriminated for the eight-vibro-tactile-tone experimental conditions (one or eight frequency bands), grouped by phoneme contrast type. Stars show the statistical significance of differences between one and eight frequency bands (corrected for multiple comparisons), with more stars indicating greater significance. Error bars show the SEM. Chance performance is marked with a dashed grey line.

Figure 4 shows discrimination for different phoneme contrasts for eight vibro-tactile tones, with either one (baseline) or eight frequency bands. For consonants, improvement was seen for voiceless fricatives pairs that differed by place of articulation (*t*(25) = 6.6, *p* < 0.001; mean effect: 18.0%; SD: 13.9%), as well as for pairs differing by voicing (*t*(25) = 12.4, *p* < 0.001; mean effect: 42.3%; SD: 17.5%), both manner and place (*t*(25) = 4.3, *p* = 0.009; mean effect: 12.5%; SD: 14.8%), and both place and voicing (*t*(25) = 10.3, *p* < 0.001; mean effect: 32.4%; SD: 16.0%). Improvement for voiceless plosive pairs that differed by place of articulation was close to significance (*t*(25) = 3.5, *p* = 0.070; mean effect: 13.5%; SD: 19.6%).

**Figure 5:**
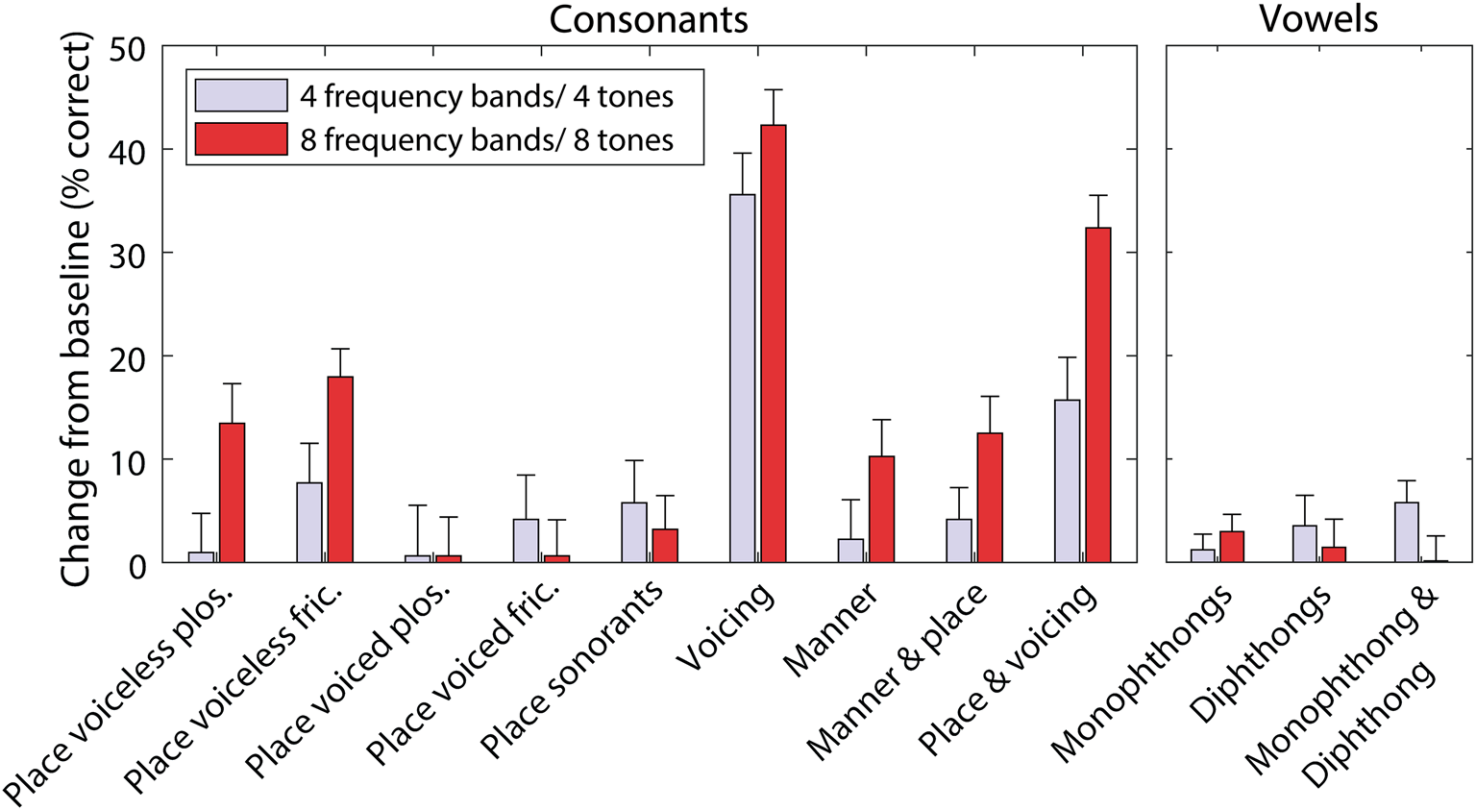
The improvement in the percentage of phoneme pairs discriminated for four or eight frequency bands compared to one frequency band, grouped by phoneme contrast type. Error bars show the SEM.

Figure 5 shows the improvement in performance compared to the one-frequency-band baseline for the four and eight frequency band conditions. No significant difference in the four and eight frequency band improvement was observed either for the consonant or vowel phoneme subgroups.

Finally, additional post-hoc uncorrected analyses were conducted to explore possible predictors of phoneme discrimination performance. The score for the eight frequency-band and eight vibro-tactile-tone condition was used as the dependent variable. No evidence of a dependence on age, wrist circumference, vibro-tactile detection thresholds at 125 Hz on the finger (measured during screening), or probe position (above, in line, or below the termination point of the ulna) was found.

## Discussion

The aim of the current study was to establish whether phoneme discrimination is improved when multiple frequency channels are available for single-site vibro-tactile stimulation on the wrist. A highly robust overall improvement in phoneme discrimination was observed with multiple frequency channels, with the largest effects seen for voicing and place contrasts. Performance was better with eight frequency bands than with four, indicating that higher resolution spectral information than has been provided in previous studies^12,14–16^ can be exploited. In the current study, the vibro-tactile tones were kept within the frequency and intensity range of the latest compact, low-powered haptic actuators. Furthermore, the audio-to-tactile vocoder signal-processing approach used can be implemented in real-time on a compact device. The eight-channel frequency-to-frequency vocoder method could therefore readily be used in a new wrist-worn haptic hearing aid.

For tactile stimulation with a single frequency channel, some phonemic information was transferred, particularly for facilitating consonant manner and place contrasts. Discrimination by consonant manner was likely achieved using differences in broadband temporal envelope patterns. However, voicing information was not well transferred through single frequency-channel stimulation. The three cognate pairs that differed by voicing were fricatives, which cannot be discriminated using strong envelope cues. For the cognate pairs with a single-frequency-channel, periodicity is likely to be a dominant voicing discrimination cue, but periodicity information is not well maintained by the amplitude envelope extraction used in the current vocoder approach.

Our results suggest that multiple frequency channels improve performance most for consonant pairs, particularly those differing by voicing alone or voicing and place. For isolated phonemes, the presence or absence of voicing (the voice bar) is conveyed primarily in frequencies below 400 Hz. The large improvement in voicing discrimination with multiple frequency channels, as compared with a single channel, is therefore likely to be due to the utilisation of frequency channels corresponding to acoustic information below 400 Hz (the lowest channel when there were four frequency channels and the lowest two channels when there were eight frequency channels). Voicing information is not accessible through lip reading and so transferring this information could have a significant functional benefit for those who receive limited acoustic information through other means^26^.

The current study showed evidence that eight frequency channels improve performance more than four for phonemes that differ by place of articulation (see Fig. 5). Discrimination of these pairs requires sufficiently high-resolution mid-to-high-frequency audio information, as place of articulation in obstruent consonants (e.g., fricatives and plosives) are signalled by the spectral pattern of the frication or burst noise at middle-to-high frequencies^27^. It is likely that this was more salient with eight frequency channels, where four of the channels are dedicated to audio frequencies above 2000 Hz, than with four frequency channels, where only two channels are dedicated to frequencies above 2000 Hz. Accurate perception of place of articulation is important, particularly when lipreading is not possible (as lipreading can be used to resolve many place differences). Furthermore, loss of access to high frequency sound (as is typical for those with sensorineural hearing-loss) can reduce the salience of place cues^28^ and many CI users also struggle to use place of articulation information because of limitations in the CI’s spectral resolution^29^. These groups may therefore both benefit from provision of these cues through tactile stimulation.

**Figure 6:**
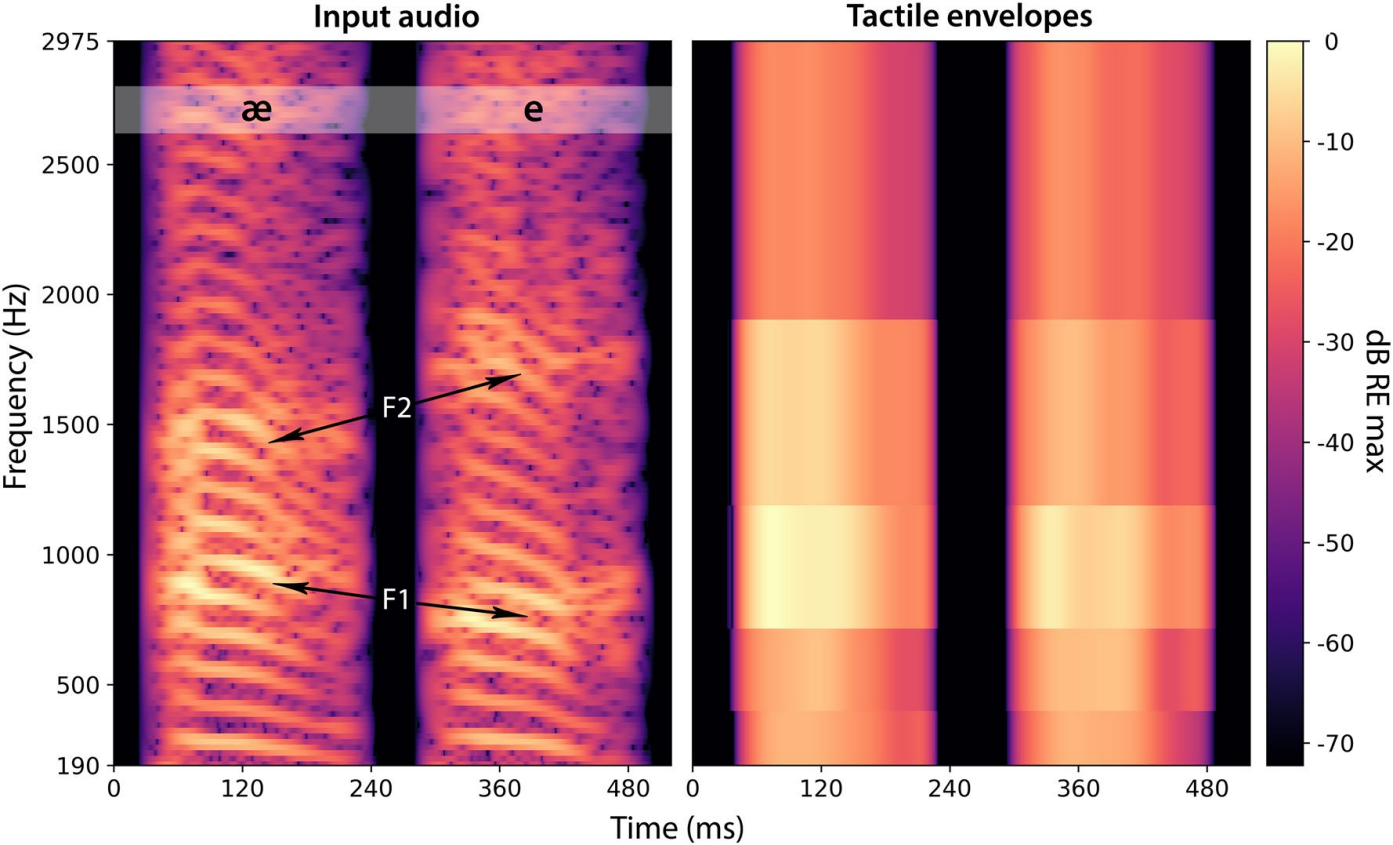
Spectrograms showing the input audio (left panel) and the tactile envelopes extracted using the eight-frequency-channel vocoder approach (right panel) for the phonemes æ and e (spoken by the male talker). The first and second formants of the input audio are marked. The upper two frequency channels and lowest channel are not shown for the tactile envelopes. The audio spectrogram sample rate was 22.05 kHz, with a window size of 1024 (Hann) and a hop size of 1 sample. The tactile spectrogram sample rate was 16 kHz, and no windowing was applied to the envelopes. Intensity is shown in decibels relative to the maximum magnitude of the STFT for the input audio and in decibels relative to the maximum envelope amplitude for the tactile envelopes. The spectrograms were generated using the Librosa Python library (version 0.10.0).

Unexpectedly, vowel discrimination was poor across all conditions tested in the current study. It may be that, even with eight frequency channels, the different frequency bands did not sufficiently separate the lowest two formants, which are important for identifying vowels. An example is shown in Fig. 6, where shifts in the first and second formant frequencies can be seen in the audio for the phonemes /æ/ and /e/, but these shifts are not well represented in the tactile signal. Future work should explore whether different frequency band allocation focused on improving the representation of formants can improve tactile vowel discrimination.

Despite the tactile system not having a highly tuned membrane to perform frequency decomposition like the cochlea, there are several means through which spectral information might have been deconstructed by participants in the current study. The first is by comparing responses across different tactile receptor types, which each have distinct frequency and temporal sensitivity^30,31^. Another is by exploiting the frequency-dependent transfer of vibration through the skin, bones, and soft tissue^32^. This could allow frequency decomposition to be achieved by assessing how excitation spreads across different receptor locations. Finally, spectral profiles might be distinguished using the firing rate of tactile nerve fibres, which are known to closely synchronise (phase lock) with the periodicity of sinusoidal vibration^33^. For stimuli with a clear dominant frequency, phase locking may occur, and, for other stimuli, the absence of phase locking may indicate the absence of a clear spectral peak.

There are important limitations to the current study. Firstly, the method used focuses on spectral or spectral-temporal speech information, and not on the ability to detect temporal boundaries of phonemes, syllables, or words (segmentation). As well as being important for tactile-only speech perception, tactile speech segmentation could be critical to improving speech perception in CI users and in those with hearing impairment, particularly in the presence of background noise. Indeed, segmentation could have played an important role in the tactile benefits observed in previous studies assessing word recognition in sentences^10,12–14^. Assessing whether speech segmentation is improved by providing additional frequency information through the eight frequency-channel audio-to-tactile vocoder approach should be a focus of future work.

The method was also limited in that it assessed discrimination rather than identification. This was done to circumvent the need for a prolonged training regime and to thereby allow relatively fast testing of basic parameters of the audio-to-tactile vocoder approach. It should be noted that discrimination is a necessary but not sufficient prerequisite for identification. While the current study controlled for absolute intensity cues (through level roving) and for broadband temporal envelope cues (which were available in the control condition), other spectro-temporal cues not relevant to identification may have facilitated discrimination. The pattern of performance improvements with multiple frequency channels across phoneme sub-groups, which are explicable based on phoneme-specific information expected to be transferred with the multi-channel vocoder approach (e.g., voicing information), suggests that phoneme-specific cues critical for identification were used. However, the current results should be interpreted with caution as the relationship between tactile phoneme discrimination and identification is not well understood.

Another limitation was that the participant group did not match the target user group for haptic hearing aids, with participants predominantly having no known hearing impairment. Several previous studies have found no differences in tactile speech performance between normal-hearing and hearing-impaired individuals (e.g.,^12,13,34,35^). However, there is evidence of increased tactile sensitivity in congenitally deaf individuals^36^, which might allow them to better exploit speech information provided through tactile stimulation. In the current study, one participant was a CI user (P14) and another had experienced persistent tinnitus for more than a decade (P2). Their results did not deviate from the other participants in the study, who reported no hearing impairment. Future work should comprehensively establish whether there are differences in tactile speech perception across potential user groups for haptic hearing aids.

Another difference between the participants in this study and the target user group is the average age. Participants were young (all under 40 years old), whereas a significant portion of the hearing-impaired community are older. In the current study, there was no evidence of a correlation between participant age (which spanned 18 years) and tactile phoneme discrimination. Furthermore, previous studies have found no effect of age on tactile intensity discrimination^17,37^ or temporal gap detection for tonal stimuli^38^. However, absolute vibro-tactile detection sensitivity^39^ and frequency discrimination^40^ has been shown to worsen with age. In future work, it will be important to establish whether older users can benefit as much from additional frequency channels as younger users.

Several important questions remain about the optimisation of the frequency-to-frequency audio-to-tactile vocoder approach. One is whether a greater number of frequency bands and vibro-tactile tones than eight can yield still better speech performance. Another is whether focusing frequency bands differently within the audio frequency range can lead to better performance (e.g., more densely sampling the frequency range around the first and second formant frequencies to try to improve vowel discrimination, as suggested above). An advantage of presenting sound information through tactile stimulation, rather than audio or CI stimulation, is that the tactile system does not have an existing frequency map for speech, which can be disrupted by frequency distortions^41^. Existing frequency compression or expansion methods for hearing aids or CIs should therefore also be tested for tactile stimulation.

An alternative approach to improving the audio-to-tactile vocoder approach might be to extract auditory features that capture key missing speech information and map them to currently unexploited tactile signal parameters. A visual inspection of auditory features extracted from the phoneme corpus used in the current study suggests that spectral crest (how tonal the signal is), spectral entropy (how dense the frequency spectrum is), spectral flux (how much the spectral shape is changing), harmonic ratio (how harmonic the signal is), and spectral centroid (the spectral centre of energy), differ across phoneme pairs where discrimination was poor. Features such as these could be mapped to, for example, frequency modulation of the vibro-tactile tones (tone frequencies in the current study were kept static) or to amplitude envelope modulations at frequencies that are not thought to be important for speech recognition but where tactile sensitivity is high (e.g., above around 30 Hz^42,43^). Alternatively, audio features could be mapped to differences in stimulation at different locations on the skin (for example, different positions around the wrist^7^ or along the arm^19^). However, it is possible that speech cues that are successfully transferred through the current eight-band vocoder approach will be masked or distorted by adding additional frequency or amplitude modulation, or by moving stimulation across sites.

Another important area for future research is the robustness of the vocoder approach to background noise. Previously, a multi-band expander technique has been used with the audio-to-tactile vocoder to enhance noise robustness^12,13^. In future work, the optimal parameters for the expander should be established and other more advanced noise-reduction techniques, such as those exploiting neural networks^44^, should be explored.

The demonstration in the current study that complex spectral information can be transferred through amplitude modulated vibro-tactile tones could have important implications for a range of other haptic devices. For example, amplitude modulated vibro-tactile tones could be used to transfer complex spatial information for other neuroprosthetic haptic devices, such as those for aiding vision^45^ or balance^46^. The approach could also be used to transfer information in other haptic feedback applications, such as medical haptic tools for needle steering^47^, remote control of research tools^48^, or human-controlled robots^49^. Additionally, it could be used to generate distinctive sensations in haptic feedback devices used in entertainment such as music^50^ or computer gaming, and to enhance virtual or augmented reality^51^.

Since tactile stimulation was last a significant focus in the hearing sciences, compact haptic actuator technology has advanced dramatically. Now, compact, low powered, high-fidelity actuators can produce intense vibration across a relatively broad frequency range where the skin is highly sensitive. This has opened an important new means through which sound information can be transferred through tactile stimulation. This study has shown that additional speech information can be transferred by exploiting these new actuator capabilities using a real-time audio-to-tactile signal-processing strategy that provides spectral information through tactile frequency differences. There is a powerful opportunity for this approach to be used in a new generation of low-cost haptic hearing aids which combine the latest haptic actuator technology with other cutting-edge technologies, such as compact long-life batteries, flexible microprocessors (which allow both advanced computation and substantially increased design flexibility), and low-latency, low-powered wireless technology (that allows the use of wireless microphones and remote data transfer^11,50^). These new haptic hearing aids could substantially improve quality-of-life for large populations of hearing-impaired individuals, including both CI users and the tens of millions of people across the world who are unable to access CI technology.

## Methods

### Participants

Participant characteristics are shown in Table 1 for the 26 adults who took part in the study. The average age was 28 years (ranging from 18 to 36 years), and there were 15 males and 11 females. All participants had normal touch perception, as assessed by a heath questionnaire and vibro-tactile detection thresholds at the fingertip (see “Procedure”). Participants were not screened for their hearing ability, but self-reported hearing status was recorded. One participant had a CI and another had persistent tinnitus in both ears that had been present for more than a decade with no known accompanying hearing loss. All other participants reported no hearing impairment. Participants were paid an inconvenience allowance of £20 for taking part.

**Table 1:** Participant characteristics.

ID	31.5 Hz thresh (m/s^2^)	125 Hz thresh (m/s^2^)	Wrist temp. (°C)	Wrist height/width (mm)	Wrist circum. (mm)	Probe site (re. ulna)	Dom. hand (L/R)	Age (yrs)	Sex (M/F)
1	0.048	0.073	31.8	41/52	160	Above	R	29	M
2	0.021	0.079	30.4	39/58	166	Below	R	36	M
3	0.036	0.063	33.0	36/51	149	Above	R	35	F
4	0.033	0.122	30.7	42/53	163	In line	R	25	M
5	0.120	0.050	30.3	38/56	164	Below	R	26	M
6	0.039	0.085	28.1	39/49	149	Below	R	30	F
7	0.033	0.069	30.2	36/48	149	Above	L	28	F
8	0.317	0.662	28.3	51/61	187	Above	R	31	M
9	0.065	0.128	31.6	49/54	182	Above	R	36	M
10	0.109	0.182	29.7	58/65	205	Above	L	29	M
11	0.038	0.036	29.9	40/57	171	Below	R	32	M
12	0.031	0.057	29.7	47/53	173	Above	R	28	M
13	0.040	0.052	31.0	46/55	174	Above	R	22	M
14	0.013	0.019	29.8	36/47	151	Above	R	22	F
15	0.045	0.088	30.3	31/44	142	Above	R	31	F
16	0.054	0.057	31.4	39/61	170	Below	R	31	F
17	0.065	0.124	28.7	33/44	139	Above	R	27	F
18	0.097	0.17	30.4	50/58	179	Above	R	26	M
19	0.030	0.057	29.8	38/47	150	Above	R	24	F
20	0.029	0.067	27.3	20/25	155	In line	L	18	F
21	0.098	0.425	32.4	20/25	159	Above	R	33	M
22	0.037	0.031	28.6	20/24	154	Above	R	27	F
23	0.015	0.070	29.7	19/27	163	Above	R	18	M
24	0.057	0.094	27.1	20/27	168	Above	R	29	M
25	0.173	0.170	29.3	22/28	176	In line	R	23	M
26	0.043	0.137	30.6	21/23	155	Above	R	22	F
Mean	0.065	0.122	30.0	35.8/25.8	163.6	–	–	28	–

The probe site is either above (towards the elbow), in line, or below (towards the hand) the terminal point of the ulna bone at the wrist (see “Procedure”).

### Stimuli

**Figure 7:**
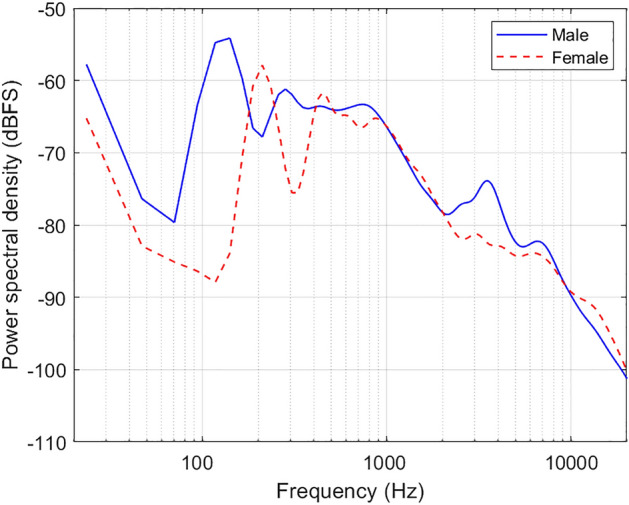
The long-term average spectrum of the male and female talker from the EHS Research Group Phoneme Corpus (based on all phonemes), with no normalisation applied.

The tactile stimulus in the experiment phase (after screening), was generated using the EHS Research Group Phoneme Corpus, which contained a southern English male and female talker saying each of the 44 UK British English phonemes. The phonemes were produced, as far as possible, in isolation. However, for some of the obstruent consonants, particularly voiced plosives, a following /ə/ was produced. For each talker, the corpus contains four tokens of each phoneme. The long-term average speech spectrum across all phonemes is shown for each talker in Fig. 7 (with no normalisation). The spectrum was calculated from the average power spectral density (Hann windowed, with a 96 kHz sample rate, an FFT length of 4096, and a hop size of 2048). The average power spectral density was Gaussian-smoothed with a 1/3 octave resolution.

The male talker had an average fundamental frequency of 145.4 Hz (SD: 12.4 Hz; ranging from 107.0 to 182.2 Hz) and the female talker had an average fundamental frequency of 208.2 Hz (SD: 14.7 Hz; ranging from 174.2 to 284.9 Hz). The fundamental frequency (estimated using a Normalized Correlation Function) and the harmonic ratio were determined using the MATLAB audioFeatureExtractor object (MATLAB R2022b). A 30-ms Hamming window was used, with a 25-ms overlap length. Samples were included in the analysis if their harmonic ratio was greater than 0.75.

A subset of 53 phoneme pairs was chosen for the phoneme discrimination task (see Table 2). Pairs were selected to ensure a wide range of phoneme contrasts, including those where discrimination is not possible using lip reading alone or using the acoustic signal alone for those with a substantial high-frequency hearing-loss (which is common in sensorineural hearing impairment). Pairs also included common vowel and consonant confusions for both high- and low-performing CI users^29^ and for users of a previous multi-channel tactile aid (Tactaid VII)^34^. This was done to maximize the functional relevance of the test set for different user groups and to include contrasts which have previously been challenging to convey through tactile stimulation.

**Table 2:** Constant and vowel pairs used in the experiment, grouped by the type of contrast.

Consonants	Contrast type	Vowels	Contrast type
*t * & *p*	Place in voiceless plosives	*ɪ * & *ɑː*	Monophthongs
*t * & *k*	Place in voiceless plosives	*iː * & *æ*	Monophthongs
*k* & *p*	Place in voiceless plosives	*ɔː * & *ɪ*	Monophthongs
*f * & θ	Place in voiceless fricatives	*ɔː * & *ɑː*	Monophthongs
*f * & *s*	Place in voiceless fricatives	*ʊ* & *ɑː*	Monophthongs
*ʃ * & *s*	Place in voiceless fricatives	*uː * & *ʌ*	Monophthongs
*d* & *b*	Place in voiced plosives	*æ * & *e*	Monophthongs
*g* & *d*	Place in voiced plosives	*ʊ * & *ɪ*	Monophthongs
*g * & *b*	Place in voiced plosives	*æ * & *ɒ*	Monophthongs
*v * & *ð*	Place in voiced fricatives	*iː * & *uː*	Monophthongs
*v * & *z*	Place in voiced fricatives	*ʌ * & *æ*	Monophthongs
*ð * & *z*	Place in voiced fricatives	*uː * & *ʊ*	Monophthongs
*l * & *r*	Place in sonorants	*ɔɪ * & *eɪ*	Diphthongs
*j * & *l*	Place in sonorants	*ɔɪ * & *aʊ*	Diphthongs
*m * & *n*	Place in sonorants	*aʊ* & *eɪ*	Diphthongs
*z* & *s*	Voicing	*ɪə* & *əʊ*	Diphthongs
*ʒ * & *ʃ*	Voicing	*ʊə * & *eɪ*	Diphthongs
θ & * ð*	Voicing	*eə* & *ʊə*	Diphthongs
*t * & *s*	Manner	*ʊ * & *eɪ*	Monophthong & diphthong
*b* & *w*	Manner	*iː * & *eɪ*	Monophthong & diphthong
*tʃ * & *ʃ*	Manner	*ɪə * & *ɒ*	Monophthong & diphthong
*ð * & *b*	Manner & place (two-feature)	*uː * & *ɪə*	Monophthong & diphthong
*k * & *s*	Manner & place (two-feature)	*əʊ* & *uː*	Monophthong & diphthong
*g* & *r*	Manner & place (two-feature)	*aʊ * & *ɑː*	Monophthong & diphthong
*v * & *s*	Place & voicing (two-feature)	*ɑː * & *eɪ*	Monophthong & diphthong
θ & *z*	Place & voicing (two-feature)	*ɔː * & *eɪ*	Monophthong & diphthong
*m * & *v*	Place & voicing (two-feature)		

The stimulus duration was matched for all pairs by fading both stimuli out with a 20-ms raised-cosine ramp, with the exception of pairs containing a diphthong or those containing the consonants */g/, /d/, /l/, /r/, /v/, /w/,* or */j/*, where production in isolation as a single phoneme (without adjacent vowel) is impossible or acoustically very different from production in running speech. The ramp reached its zero-amplitude point at the end of the shortest stimulus (defined as the point at which the signal had dropped below 1% of its absolute maximum). This ensured that, for these pairs, discrimination could not be achieved by comparing the durations of the stimuli.

The audio was converted to tactile stimulation using a vocoder method similar to that used in previous studies^12–16^. The signal intensity was first normalised following ITU P.56 method B^52^. It was then downsampled to a sampling frequency of 16,000 Hz (matching that available through many hearing aids and other compact real-time audio devices). Following this, the signal was passed through a 512th-order FIR filter bank with one, four, or eight frequency bands (depending on the experimental condition) between 50 and 7000 Hz. This frequency range was selected to follow ITU-T G.722^53^, and focused on the range in which there is substantial speech energy (see Fig. 7 and^54^). It is also similar to the range used in previous studies that have shown large improvements in speech-in-noise performance^14^ and sound localisation^15,16^ in CI users. For conditions with four or eight frequency bands, the frequency bands were equally spaced on the auditory equivalent rectangular bandwidth scale^55^. Next, the amplitude envelope was extracted for each frequency band using a Hilbert transform and a zero-phase 6th order Butterworth low-pass filter, with a corner frequency of 23 Hz. This filter was designed to focus on the envelope modulation frequencies most important for speech recognition^42^. These amplitude envelopes were then used to modulate the amplitudes of one, four, or eight fixed-phase vibro-tactile tonal carriers (depending on the experimental condition).

For the one vibro-tactile-tone and one frequency-band condition, the vibro-tactile tone frequency was set to 170 Hz to match the frequency at which vibration output is maximal for many compact haptic actuators. For the four-vibro-tactile-tone conditions, the tones were at 138, 170, 210, and 259.5 Hz. The tone frequency range was focused around 170 Hz, and the frequencies were spaced so that each tone could be discriminated, based on data at the palmer forearm^24^ (no tactile frequency discrimination data for the wrist is known to the authors). For the eight-vibro-tactile-tone conditions, the tones were at 94.5, 116.5, 141.5, 170, 202.5, 239, 280.5 and 327.5 Hz. These were more tightly spaced based on frequency discrimination thresholds at the dorsal forearm^25^ in order to remain within the frequency range that can be reproduced by compact, low-powered haptic actuators that are suitable for a wrist-worn device (either specialist wideband actuators or multiple actuators used together with a frequency crossover filter). It should be noted that the available data suggests that both estimates of frequency discrimination are conservative, as the wrist is thought to have similar frequency discrimination to the finger^56^, which has better frequency discrimination thresholds than the forearm^24^.

A frequency-specific gain was applied to each vibro-tactile tone so that it was equally exciting, based on tactile detection thresholds^24^. For the four vibro-tactile tones, the gains were 9.6, 5.8, 0.4, and 0 dB, respectively, and, for the eight vibro-tactile tones, the gains were 13.8, 12.1, 9.9, 6.4, 1.6, 0, 1.7, and 4 dB, respectively. The tactile stimuli generated were scaled to have an equal overall amplitude in RMS, giving a nominal level of 141.5 dB ref 10^–6^ m/s^2^ (1.2 G), which is an intensity that can be produced by a range of compact, low-powered shakers. This stimulus level was roved by 3 dB around the nominal level (with a uniform distribution) to ensure that no discrimination cues based on absolute intensity were available. To mask any audio cues that might be used to discriminate the tactile stimuli, a pink noise was presented at 60 dBA.

### Apparatus

Participants were seated in a vibration isolated, temperature-controlled room (mean temperature: 23 °C; SD: 0.45 °C). The room temperature and the participant’s skin temperature were measured using a Digitron 2022 T type K thermocouple thermometer. The thermometer was calibrated following ISO 80601-2-56:2017^57^. For calibration, the thermocouple was submerged and calibrated using three mercury glass bead thermometers (ASTM 90C, ASTM 91C, and ASTM 92C), which covered different temperature ranges. These thermometers were calibrated by C.I.S Calibration Laboratories (Leicestershire, UK). For cold temperatures (5 °C to 20 °C), a Grant GD120 water bath with a Grant ZD circulation unit and Grant C2G refrigeration unit was used, and for warmer temperatures (25 °C to 50 °C), a Grant Y6 water bath with a Grant VF circulation unit was used.

For the screening vibro-tactile detection threshold measurements, a HVLab Vibro-tactile Perception Meter^58^ was used that conformed to ISO-13091-1:2001^59^. The Vibro-tactile Perception Meter had a circular probe with a 6-mm diameter and a rigid surround. The probe gave a constant upward force of 1N. A downward force sensor was built into the surround, and the force applied was displayed to the participant. The sensor was calibrated using Adam Equipment OIML calibration weights. The vibration intensity was calibrated using the Vibro-tactile Perception Meter’s built-in accelerometers (Quartz Shear ICP, model number: 353B43) and a Brüel & Kjær (B&K) Type 4294 calibration exciter.

**Figure 8:**
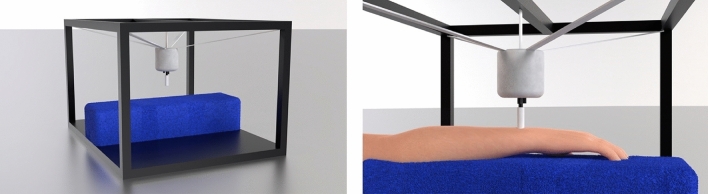
A 3D rendered image of the EHS Research Group haptic stimulation rig used in the current study. The left image shows the set up with no arm in place and the shaker and probe free hanging. The right image shows a close view of the rig with the arm in place and the shaker probe contacting the wrist.

In the experiment phase, a custom EHS Research Group haptic stimulation rig was used. This consisted of a Ling Dynamic Systems V101 shaker, with a 3D printed circular probe (Verbatim Polylactic Acid material) that had a 10-mm diameter and no rigid surround. The shaker was driven using a MOTU UltralLite-mk5 sound card, RME QuadMic II preamplifier, and HV Lab Tactile Vibrometer power amplifier. The shaker was suspended using an adjustable elastic cradle from an aluminium strut frame (see Fig. 8). The probe applied a downward force of 1N, measured using a B&K UA-0247 spring balance. The rig allowed the vibration probe to contact the dorsal wrist, with the palmar forearm resting on a 95 mm thick foam surface. The vibration output was calibrated using a B&K 4533-B-001 accelerometer and a B&K type 4294 calibration exciter. All stimuli had a total harmonic distortion of less than 0.1%.

Masking audio was played from the MOTU UltralLite-mk5 sound card through Sennheiser HDA 300 headphones. The audio was calibrated using a B&K G4 sound level meter, with a B&K 4157 occluded ear coupler (Royston, Hertfordshire, UK). Sound level meter calibration checks were carried out using a B&K Type 4231 sound calibrator.

The EHS Research Group Phoneme Corpus used in the experimental phase was recorded in the anechoic chamber at the Institute of Sound and Vibration Research. The audio was recorded using a B&K 4189 microphone, B&K 2669 preamplifier, B&K Nexus 2690 conditioning amplifier, and RME Babyface Pro soundcard (with a 96 kHz sample rate and a bit depth of 24 bits). The microphone was 0.3 m from the talker’s mouth.

### Procedure

Each participant completed the experiment in a single session lasting approximately 2 h. First, written informed consent was obtained from all the participants. Participants then completed a screening questionnaire to ensure they (1) did not suffer from any conditions that might affect their sense of touch (e.g., diabetes), (2) had not had any injury or surgery on their hands or arms, or (3) had not been exposed to severe or long periods of hand or arm vibration in the previous 24 h. Next, the wrist dimensions were measured at the site at which the participant would normally wear a wristwatch (this was also where the probe contacted the wrist in the experiment phase). The participant’s skin temperature was then measured on the index fingertip of their dominant hand. Participants were only allowed to continue the screening when their skin temperature was between 27 and 35 °C. Following this, vibro-tactile detection thresholds were measured at the index fingertip following BS ISO 13091-1:2001^59^. During the threshold measurements, participants applied a downward force of 2N (monitored by the participant and experimenter using the HVLab Vibro-tactile Perception Meter display). Participants were required to have touch perception thresholds in the normal range (< 0.4 m/s^2^ RMS at 31.5 Hz and < 0.7 m/s^2^ RMS at 125 Hz), conforming to BS ISO 13091‑2:2021^60^. The fingertip was used as there is not sufficient normative data available at the wrist. If participants passed the screening phase, they moved to the experiment phase.

In the experiment phase, participants were seated in front of the EHS Research Group haptic stimulation rig (see Fig. 8), with the palmar forearm of their dominant arm resting on a foam surface and the vibro-tactile stimulation probe contacting the centre of the dorsal wrist. The probe was positioned where the participant reported they would normally wear a wristwatch. This meant that the probe was either slightly above (towards the elbow), in line, or slightly below (towards the hand) the terminal point of the ulna bone at the wrist (see Table 1).

The participants completed a three-interval, three-alternative forced-choice phoneme discrimination task. The inter-stimulus interval was 250 ms. Each trial used a pair of phonemes from a single talker (see “Stimulus”). One phoneme from the pair was presented in one of the three intervals and the other phoneme was presented in the other two intervals. Which phoneme of the pair was presented once, and which was presented twice was randomised. The order of intervals was randomised and the participant’s task was to select the interval containing the phoneme presented only once via a key press. Participants were instructed to select the vibration that felt different from the others (i.e., the odd one out), but to ignore the overall intensity of each vibration. After each trial, visual feedback was given indicating whether the response was correct or incorrect.

The percentage of phonemes correctly discriminated was measured in five conditions, each with different tactile stimulation parameters: (1) with one frequency band and one vibro-tactile tone (1FB1T), (2) with one frequency band and four vibro-tactile tones (1FB4T), (3) with four frequency bands and four vibro-tactile tones (4FB4T), (4) with one frequency band and eight vibro-tactile tones (1FB8T), and (5) with eight frequency bands and vibro-tactile tones (8FB8T). For each condition, all phoneme pairs were tested for both the male and female talker. For each talker, two repeats of each phoneme pair were tested, with the phoneme sample randomly selected from the four available for each phoneme. The order of conditions was randomised for each phoneme pair repeat.

The experimental protocol was approved by the University of Southampton Faculty of Engineering and Physical Sciences Ethics Committee (ERGO ID: 68477). All research was performed in accordance with the relevant guidelines and regulations.

### Statistics

The percentage of phonemes correctly identified was calculated for each condition for the male and female talkers. Primary analysis consisted of three two-tailed *t*-tests. These compared conditions 1FB4T to 4FB4T, 1FB8T to 8FB8T, and 4FB4T-1FB4T to 8FB8T-1FB8T. These tests had a Bonferroni-Holm correction^61^ for multiple comparisons applied (correction for three tests).

Next, secondary analyses were conducted. This included two two-way RM-ANOVAs, which were run on the differences between multiple frequency band conditions, one for the vowels and one for the consonants. A third three-way RM-ANOVA was run on the baseline conditions (the conditions with one frequency band). For the RM-ANOVAs, no evidence of a breach of the assumption that data were normally distributed was found in Kolmogorov–Smirnov or Shapiro–Wilk tests and, for the baseline conditions, Mauchly’s test indicated that the assumption of sphericity had not been violated. The RM-ANOVAs used an alpha level of 0.05.

In addition to the three RM-ANOVAs, two-tailed *t*-tests were run assessing the differences between 4FB4T and its baseline (1FB4T) and 8FB8T and its baseline (1FB8T) for each of the phoneme pair subgroups (see Table 2). The differences between the effects observed for the four and eight frequency band conditions were also tested for each phoneme pair subgroup. All these secondary analyses had a Bonferroni-Holm multiple comparisons correction applied (correction for 51 tests, which included the tests done in the primary analysis).

Finally, three Spearman correlations were run between the 8FB8T condition score and the screening vibro-tactile detection threshold at 125 Hz, participant age, and wrist circumference (see Table 1). These variables were thought to have the most potential to correlate with phoneme task performance. In addition, a one-way RM-ANOVA with the factor ‘Probe position’ (above, in line, or below the termination point of the ulna) was run. For each of these exploratory tests it was hypothesised that no effect would be found, so no correction for multiple comparisons was applied.

## Data Availability

The datasets generated and analysed during the current study are available in the University of Southampton’s Research Data Management Repository at: 10.5258/SOTON/D2739.
